# Comparative Analysis of Demographic Characteristics, Management, and Outcomes in Primary Versus Secondary Spontaneous Pneumothorax

**DOI:** 10.7759/cureus.65216

**Published:** 2024-07-23

**Authors:** Gamal Abdelshafy Ibrahim Farag, Mokhles Abdelfadil Ibrahim Zineldin, Ramadan Shawky Abd Alaziz Al Awady, Ahmed Bedeir Abd El Salam, Mohamed Attia Elkahely

**Affiliations:** 1 Cardiothoracic Surgery Department, Damietta Faculty of Medicine, Al-Azhar University, Damietta, EGY; 2 Chest Diseases Department, Damietta Faculty of Medicine, Al-Azhar University, Damietta, EGY; 3 Radiology Department, New Damietta Hospital, Al-Azhar University, Damietta, EGY; 4 Cardiothoracic Surgery, Faculty of Medicine for Girls, Al-Azhar University, Cairo, EGY

**Keywords:** demographics, management approaches, smoking, secondary spontaneous pneumothorax, primary spontaneous pneumothorax

## Abstract

Objectives: This study aimed to explore the differences between primary spontaneous pneumothorax (PSP) and secondary spontaneous pneumothorax (SSP) in demographic and clinical features, management trends, and outcomes, alongside assessing recurrence risk factors in spontaneous pneumothorax (SP) patients.

Methods: This retrospective cohort study at New Damietta Hospital, Al-Azhar University, examined data from adults diagnosed with SP, differentiating between PSP and SSP types based on clinical and radiological criteria, to analyze demographics, clinical characteristics, management strategies, and outcomes.

Results: In a study of 170 patients, 42.94% were diagnosed with PSP and 57.06% with SSP, showing significant differences in age distribution (P=0.042) and smoking habits (P<0.001 for both tobacco and cannabis). Management approaches varied, with conservative methods more common in PSP (15.07%) and surgical interventions following intercostal tube (ICT) drainage significantly higher in SSP (40.21%, P=0.001). Length of hospital stay (LOS) and recurrence rates were significantly higher in SSP than PSP (P<0.001 for LOS; P=0.001 for recurrence), with postoperative complications and in-hospital mortality occurring exclusively in SSP (P=0.054 for complications, P<0.001 for mortality). Risk factors for recurrence included older age, presence of blebs/bullae (P<0.001), and lower hemoglobin and hematocrit levels (P=0.009 and P=0.008, respectively), with thoracic drainage duration longer in recurrent cases (P=0.008). Smoking status significantly impacted recurrence risk, with current smokers showing a higher risk compared to never-smokers (P=0.012).

Conclusions: This study highlights significant demographic, clinical, and management differences between primary and secondary spontaneous pneumothorax, underscoring the importance of tailored treatment strategies to improve patient outcomes. Key findings include the impact of smoking status on recurrence risk and the necessity for individualized management plans, especially in SSP patients who exhibit higher rates of recurrence, longer hospital stays, and greater morbidity.

## Introduction

Pneumothorax, a disorder characterized by the collection of air in the pleural space, can occur spontaneously (spontaneous pneumothorax, i.e., SP) without trauma, known as primary (primary SP, i.e., PSP) in individuals without lung disorders or secondary (secondary SP, i.e., SSP) in those with conditions like chronic obstructive pulmonary disease (COPD) or as a recent complication of coronavirus disease 2019 (COVID-19) [1,2]. Despite advancements in treatment, SP, especially PSP, presents a clinical challenge, requiring nuanced management strategies to cater to diverse patient needs and clinical scenarios [3]. SP, with PSP being the most prevalent form, constitutes a significant global health challenge, featuring varying annual incidences of 18-28 cases per 100,000 in men and 1.2-6 cases per 100,000 in women for PSP, and approximately 6.3 and two cases per 100,000 for SSP among men and women, respectively [4-7]. The differentiation between PSP and SSP is crucial for tailored treatment, although distinctions are increasingly nuanced due to the discovery of subtle lung abnormalities in presumed PSP cases, suggesting a potential future revision of the classification systems [8,9].

PSP predominantly affects individuals aged 10 to 30, while SSP typically occurs in those aged 60 to 64, often presenting as a life-threatening condition due to pre-existing compromised lung function. This age-related incidence disparity underscores the need for further investigation into etiological factors, with smoking and occupational exposures identified as significant risk factors for SSP, suggesting implications for public health measures [5,10,11]. Tobacco smoking emerges as the primary risk factor for PSP, with studies showing significantly increased risks for smokers compared to non-smokers, alongside a dose-response relationship with the number of cigarettes smoked [12]. Cannabis use further compounds this risk, likely due to similar lung damage and the effects of coughing or Valsalva maneuvers specific to smoking cannabis [13]. Additionally, PSP incidence has correlations with environmental factors like atmospheric pressure, air pollution, height, and a low body mass index (BMI), suggesting a multifaceted risk profile [14,15].

Pneumothorax poses a significant clinical challenge, with recurrence rates varying widely from 8% to 74%, complicating the determination of risk and treatment strategy. Risk factors for recurrence in PSP include female gender, low body weight, smoking, and height in men, along with radiological indicators like bullae on CT scans [16]. Despite the high variability in recurrence rates, with figures between 17% and 54%, no definitive predictors for PSP recurrence have been established. SSP shows even higher recurrence rates and worse outcomes compared to PSP. Preventative measures after recurrence vary, with some guidelines recommending interventions after the second SSP episode, while others advocate for immediate action post-first episode. The effectiveness of treatments such as medical pleurodesis and surgical interventions varies, with recurrence rates decreasing significantly post-intervention [4,17-19].

While PSP commonly affects younger, healthier individuals, recommending initial conservative treatments or interventions like needle aspiration (NA) or chest tube drainage, SSP occurs in an older demographic with pre-existing lung conditions, often necessitating more complex management approaches due to higher morbidity, mortality, and recurrence rates. Management guidelines from organizations like the American College of Chest Physicians (ACCP) and the British Thoracic Society (BTS) emphasize surgical intervention in cases of non-resolving pneumothorax or recurrent episodes, with video-assisted thoracoscopic surgery (VATS) showing favorable outcomes in reducing recurrence rates and hospital stays [20,21]. However, the management of prolonged air leaks remains a significant consideration, with options ranging from continued drainage to surgical interventions, reflecting the need for individualized treatment plans to optimize patient outcomes [22]. In this study, we aimed to investigate the differences between PSP and SSP in terms of demographic and clinical characteristics, management trends, and postoperative outcomes. In addition, we aimed to assess the risk factors of recurrence in patients with SP.

## Materials and methods

Study design and setting

This retrospective cohort study was conducted at our hospital (New Damietta Hospital), analyzing data from consecutive patients diagnosed with SP who were hospitalized between January 2020 and December 2022. The study received approval from the Institutional Review Board (IRB) and Ethical Committee of New Damietta Hospital, Damietta Faculty of Medicine, Al-Azhar University, Damietta, Egypt (Approval No.: 00012367).

Participants

The study population consisted of adults (≥16 years) admitted with a diagnosis of SP, identified through a review of records from the cardiothoracic, radiology, and chest departments. Cases were categorized into PSP and SSP based on established clinical and radiological criteria. Exclusion criteria included traumatic or iatrogenic pneumothorax, patients under 16 years, and incomplete data records. In addition, patients with ischemic heart disease (IHD), chronic kidney disease (CKD), and chronic liver disease (CLD), were excluded.

Data collection

**Figure 1:**
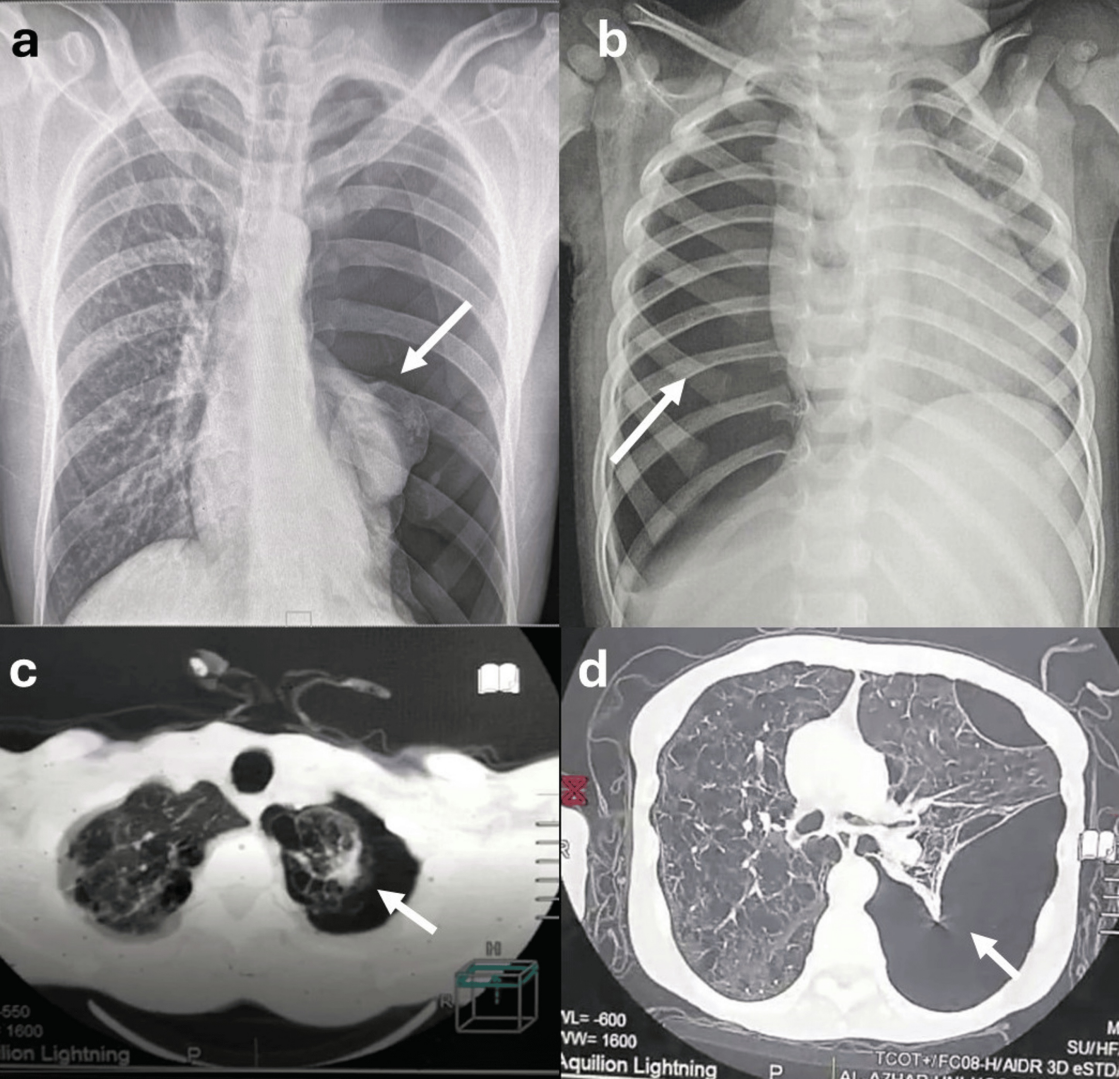
(a) X-ray of the chest (arrow indicates left side tension primary spontaneous pneumothorax); (b) X-ray of the chest (arrow indicates right side tension primary spontaneous pneumothorax); (c and d) CT of the chest (arrows indicate left side secondary spontaneous pneumothorax).

Patient records from the cardiothoracic, radiology, and chest departments were reviewed. Data on symptoms, type of pneumothorax, laterality, length of stay (LOS), management, and outcomes were collected. Demographic characteristics (age, sex distribution, smoking habits, occupation), BMI, laboratory profile (complete hemogram, coagulation tests, liver and renal function tests), and radiological data (chest x-rays, and chest CT results), were recorded (Figure 1).

**Figure 2:**
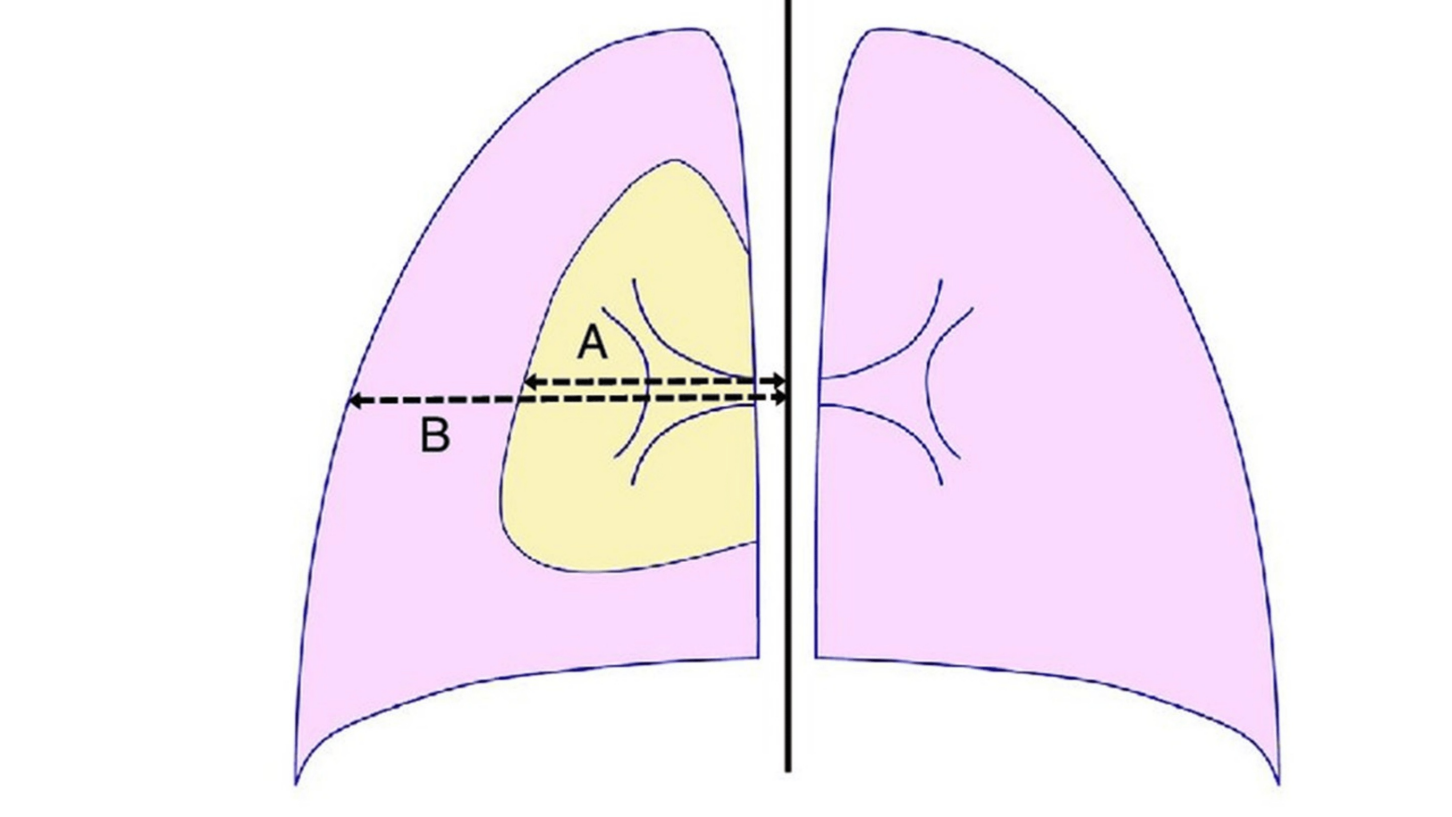
Light's index for estimating the size of pneumothorax. (A) Lung diameter and (B) hemithorax diameter (both measured at the level of pulmonary hilum). Source: [4]

The study analyzed radiological findings from X-ray and CT scans, focusing on the detection of bullae/blebs, pneumothorax laterality, and quantification of lung height at maximal inspiration. The size of the pneumothorax was calculated employing Light's index (Figure 2), defined as the percentage reduction in lung volume, determined by the formula: {1 - (L^3/HT^3)} × 100, where L represents the lung diameter and HT the hemithorax diameter, both measured at the level of the pulmonary hilum.

Treatment approaches for SP were recorded, including conservative measures, NA, intercostal drainage (ICD), and the duration of drain placement. Surgical interventions included options such as open thoracotomy (OT) and VATS, alongside specific parenchymal resections (bullectomy, segmentectomy, lobectomy) and pleurodesis methods (mechanical or chemical). Management strategies for SP were tailored according to clinical presentation, including symptomatology and hemodynamic stability, as well as the pneumothorax's size, etiology, and whether it represents an initial or subsequent episode. Divergent approaches between leading guidelines, such as those of the BTS and ACCP, were noted, particularly in the criteria for classifying a pneumothorax as large BTS uses a threshold of >2 cm from the lung margin to the chest wall at the hilum level, whereas ACCP specifies ≥3 cm from the lung apex to the thoracic cupola.

In this study, treatment modalities for SP encompassed oxygen inhalation (OI), chest drainage via a chest tube (16-24 Fr), and surgical interventions including OT or VATS with wedge resection, employed in cases of treatment failure with OI and simple ICD, or recurrence. Management decisions were guided by the size of the pneumothorax, categorized using Light’s index: conservative management for pneumothorax less than 15%, NA for 15%-30%, and ICD for those exceeding 30%. Patients exhibiting prolonged air leakage or recurrence were considered for surgery, whereas those deemed unsuitable for surgery underwent pleurodesis using tetracycline, bleomycin, or talc powder.

Outcomes

Outcomes assessed included hospital stay duration, in-hospital mortality, recurrence rates, and the incidence of postoperative complications, namely wound infections and prolonged air leaks. LOS refers to the total duration of a patient's hospital stay for a single episode of care, commencing from the initial day of admission to the day of discharge. Recurrence rate was defined as the occurrence of a new pneumothorax necessitating further intervention, such as the placement of a chest tube or undergoing repeat surgery through either VATS or OT. In-hospital mortality is identified as any death occurring during the hospitalization period for any reason. A cure is achieved when there is a complete cessation of air leakage and no recurrence of SP is observed.

Surgical approach

The surgical intervention emerged as the most effective strategy for mitigating the recurrence of SP, particularly when patient comorbidities permitted. The primary aim of these procedures was to manage the air leak and facilitate the formation of pleural adhesions. Typically, the surgical regimen involved a combination of bullectomy and pleurodesis-either via talc application, pleurectomy, or pleural abrasion.

SP treatments utilized both OT and VATS, with open procedures targeting the fourth or fifth intercostal space. Pleurodesis varied between mechanical methods, such as pleural abrasion with or without apical pleurectomy, and chemical approaches involving tetracycline, bleomycin, or talc powder. Bullectomies were performed upon identification of blebs. To facilitate postoperative recovery, one or two chest tubes were inserted for drainage, allowing patients to be discharged a day after the tubes' removal, contingent on the absence of residual pneumothorax on subsequent chest X-rays.

Complications, categorized into respiratory issues or postoperative bleeding, were meticulously documented. Respiratory complications ranged from atelectasis requiring bronchoscopy, empyema, prolonged mechanical ventilation, and acute respiratory distress syndrome (ARDS), to air leaks persisting beyond five days. Hemothorax necessitating surgical intervention was identified as postoperative bleeding. Additionally, data on in-hospital LOS and recurrence rates were collected retrospectively, providing comprehensive insight into the outcomes and challenges of surgical management for SP.

Statistical analysis

Baseline characteristics of the study participants were systematically analyzed using descriptive statistics to outline the demographic and clinical profiles. To compare patient characteristics and assess treatment outcomes, statistical analyses were conducted utilizing the Chi-square test, Fisher’s exact test, and independent samples t-tests as suited to the data type. Categorical variables were summarized as frequencies and percentages, while continuous variables adhering to a normal distribution were expressed as mean values ± standard deviations. The study further employed univariate logistic regression analysis to explore risk factors of recurrence, specifically examining variables such as sex, laterality of the pneumothorax, and smoking status. Statistical analysis was conducted using the IBM SPSS Statistics for Windows, Version 25 (IBM Corp., Armonk, NY) with significance levels set at p-values < 0.05 to determine meaningful differences.

## Results

Demographic and clinical characteristics

In this study involving 170 patients, 73 (42.94%) were diagnosed with PSP, and 97 (57.06%) with SSP. The age distribution showed PSP patients typically ranged from 35.8±12.5 years, whereas SSP patients were older, ranging from 42.4±25.2 years, with a statistically significant difference (P=0.042). The sex distribution was predominantly male, with PSP having one female and 72 males (98.6% male), and SSP consisting entirely of males, with no statistically significant difference (P=0.183). Smoking habits differed significantly between the groups; in the PSP group, 41 patients (56.2%) reported tobacco smoking and 29 (39.7%) cannabis smoking. Contrastingly, in the SSP group, a higher prevalence was observed with 92 patients (94.8%) reporting tobacco smoking and 56 (57.7%) cannabis smoking, indicating a significant association between smoking and SSP (P<0.001 for both tobacco and cannabis smoking). COPD was reported in 37 (38.14%) patients in the SSP group; however, in the PSP group, there were no patients with COPD (p<0.001). Regarding the laterality, the right side was more common in the SSP group compared to PSP (57.73% vs. 45.21%), while the left side was more common in the PSP than in SSP (52.05% vs. 41.24%), respectively, as shown in Table 1.

**Table 1:** Demographic and clinical characteristics. COPD, chronic obstructive pulmonary disease; SP, spontaneous pneumothorax; PSP, primary spontaneous pneumothorax; SSP, secondary spontaneous pneumothorax.

Parameters		PSP (n=73)	SSP (n=97)	P-value
Age		35.8±12.5	42.4±25.2	0.042
Gender	Male	72 (98.63%)	97 (100%)	0.183
	Females	1 (1.37%)	0 (0.0%)	
Smoking	Tobacco smoking	41 (55%)	92 (95%)	< 0.001
	Cannabis smoking	29 (39%)	56 (57%)	
COPD		0 (0.0%)	37 (38.14%)	< 0.001
Laterality	Right	33 (45.21%)	56 (57.73%)	0.223
	Left	38 (52.05%)	40 (41.24%)	
	Bilateral	2 (2.74%)	1 (1.03%)	

Management approaches

In the management of PSP and SSP among 170 patients, the approaches varied significantly (P=0.001). For PSP, conservative management was applied in 11 cases (15.07%), intercostal tube (ICT) drainage in 62 cases (84.93%), and surgical intervention following ICT in seven cases (9.59%). In contrast, SSP saw a markedly different management strategy: conservative methods were used in only three cases (3.09%), ICT drainage in 94 cases (96.91%), and a significantly higher proportion underwent surgery after ICT, totaling 39 cases (40.21%). Non-surgical treatment (either drainage with/without pleurodesis or observation only) was applied in 6.85% of PSP cases and 11.34% of SSP cases (P=0.321), and surgical interventions (with/without pleurodesis) were performed in 0% of PSP patients and 3.1% of SSP patients (P=0.260), as shown in Table 2.

**Table 2:** Management approaches. COPD, chronic obstructive pulmonary disease; ICT, intercostal chest tube; PSP, primary spontaneous pneumothorax; SSP, secondary spontaneous pneumothorax.

Management	PSP (n=73)	SSP (n=97)	P-value
Conservative	11 (15.07%)	3 (3.09%)	0.001
ICT	62 (84.93%)	94 (96.91%)	
Surgical after ICT	7 (9.59%)	39 (40.21%)	
Non-surgical treatment (drainage with/without pleurodesis or observation only)	5 (6.85%)	11 (11.34%)	0.321
Surgery (with/without pleurodesis)	0 (0.0%)	3 (3.1%)	0.260

Outcomes

The length of hospital stay was notably shorter for PSP patients, averaging 7 ± 2 days, compared to SSP patients, who stayed for an average of 14 ± 2 days (P<0.001). Recurrence rates also varied significantly, with PSP experiencing a lower rate of five (6.85%) cases compared to 35 (36.08%) cases in SSP (P=0.001). Postoperative complications, including wound infections and prolonged air leaks (more than five days), were more common in SSP (9.28% and 8.25%, respectively) than in PSP (1.37% and 4.11%, respectively), though the difference approached but did not reach statistical significance (P=0.054). In-hospital mortality was absent in PSP patients but occurred in 2.06% (two cases) of SSP patients, revealing a significant difference (P<0.001), as shown in Table 3.

**Table 3:** Outcomes. PSP, primary spontaneous pneumothorax; SSP, secondary spontaneous pneumothorax; LOS, length of stay.

Parameters		PSP (n=73)	SSP (n=97)	P-value
Length of stay (LOS)		7 ± 2 days	14 ± 2 days	< 0.001
Recurrence		5 (6.85%)	35 (36.08%)	0.001
Postoperative complication	Wound Infection	1 (1.37%)	9 (9.28%)	0.054
	Air leak more than 5 days	3 (4.11%)	8 (8.25%)	
In-hospital mortality		0 (0.0%)	2 (2.06%)	< 0.001

Risk factors of recurrence

In this study, 40 (23.5%) experienced recurrence. Age was a significant factor, with recurrent cases being older (29.6 ± 8.7 years) compared to those without recurrence (24.3 ± 8.1 years), presenting an odds ratio (OR) of 0.96 (95% CI: 0.93, 0.99; P=0.011). The presence of blebs/bullae on imaging was strongly associated with recurrence, exhibiting an OR of 5.34 (95% CI: 2.81, 10.23; P<0.001). Hematologic parameters such as hemoglobin and hematocrit levels were significantly lower in the recurrence group, with values suggesting an association with recurrence rates (P=0.009 and P=0.008, respectively). The analysis of smoking habits indicated that never-smokers had a lower risk of recurrence compared to current smokers, with significant differences observed (P=0.012 for smokers and P=0.039 for ex-smokers).

Treatment approaches varied, with the majority of patients (91.7%) undergoing thoracic drainage. Notably, the duration of thoracic drainage was significantly longer in patients with recurrent SP, averaging 5.5 ± 2.4 days, compared to 2.7 ± 1.8 days for those without recurrence (OR: 1.20, 95% CI: 1.04, 1.37; P=0.008). This indicates a higher complexity or severity in cases that led to recurrence. Additionally, the study highlighted a critical evaluation of treatment outcomes, where observation alone was associated with a higher recurrence (64.3% among observed patients), contrasting sharply with the success of thoracic drainage, which demonstrated a protective effect against recurrence with an OR of 0.19 (95% CI: 0.08, 0.46; P<0.001), as shown in Table 4.

**Table 4:** Risk factors of recurrence. OR, odds ratio; PTX: pneumothorax.

Variable		Total	No-recurrence	Recurrence	OR (95% CI)	P-value
N		170	130	40	-	-
Men (%)		169 (99%)	129 (76%)	40 (34%)	0.71 (0.38, 1.29)	0.259
Age (mean)		25.9 ± 8.5	24.3 ± 8.1	29.6 ± 8.7	0.96 (0.93, 0.99)	0.011
Body mass index		21.3 ± 2.9	21.6 ± 2.8	20.9 ± 3	0.92 (0.83, 1.01)	0.079
Smokers	Never-smokers (%)	12 (7.1%)	9 (75%)	3 (25%)	Reference	-
	Smokers (%)	133 (78.2%)	56 (42.1%)	77(57,9%)	0.51 (0.30, 0.87)	0.012
	Ex-smokers (%)	25 (14.7%)	17 (68%)	8 (32%)	0.23 (0.06, 0.93)	0.039
	Packs-year	9.4 ± 6.9	9.8 ± 6.6	9 ± 7.2	0.95 (0.91, 0.99)	0.566
Right-side PTX		89 (53.2%)	48 (53.5)	41 (46.5)	1.32 (0.80, 2.17)	0.274
Blebs/bullae		97 (57%)	38 (39.2)	59 (60.8)	5.34 (2.81, 10.23)	<0.001
Light’s index		44.1 ± 27.6	48 ± 26	40 ± 28	0.99 (0.98, 0.99)	0.004
Hemoglobin (g/dL)		13.9 ± 1.2	14.1 ± 1.1	13.7 ± 1.2	0.75 (0.60, 0.93)	0.009
Hematocrit (%)		43.4 ± 3.4	44 ± 3.2	42.9 ± 3.4	0.90 (0.84, 0.97)	0.008
Leukocytes		9.8 ± 3.2	10.5 ± 3.3	9.2 ± 3	0.87 (0.81, 0.95)	0.001
Platelets		250.1 ± 59	258.4 ± 61.3	244 ± 56.2	0.99 (0.99, 1.00)	0.096
Treatment-observation (%)		14 (8.2%)	5 (35.7%)	9 (64.3%)	Reference	-
Thoracic drainage (%)		156 (91.7%)	85 (54.6)	71 (45.4)	0.19 (0.08, 0.46)	<0.001
Drainage duration (days)		4.1 ± 2.1	2.7 ± 1.8	5.5 ± 2.4	1.20 (1.04, 1.37)	0.008

## Discussion

The comparative analysis between PSP and SSP in our study revealed distinct differences in patient demographics, clinical characteristics, and outcomes that are crucial for tailoring management strategies. Notably, 57% of patients presented with SSP, attributed mainly to occupational exposures such as air pollution from dusty jobs, including furniture work and challenges faced by fishermen due to adverse weather, thus leading to a higher SSP prevalence in our cohort. This finding contrasts with previous studies by Onuki et al. and Erez et al., where PSP was more prevalent, indicating the importance of environmental and occupational factors in the incidence of pneumothorax [11,23]. Our results underscore the significant impact of smoking and specific occupations on the development of SSP, reinforcing the established link between these risk factors and pneumothorax as supported by research from Hobbs et al., Onuki et al., and Erez et al. [10,11,23].

Demographic analysis further highlighted differences in age distribution between PSP and SSP patients, with PSP predominantly affecting younger individuals compared to SSP, mirroring findings by Sadikot et al. [24]. This age disparity suggests underlying pathophysiological differences between the two groups, with PSP often linked to the rupture of subpleural blebs in otherwise healthy lungs and SSP associated with pre-existing lung pathologies. The gender distribution did not show a significant difference, aligning with Onuki et al. and Brown et al., indicating that spontaneous pneumothorax affects both genders [11,25]. These insights into the demographic and clinical characteristics of PSP and SSP patients highlight the necessity for clinicians to consider individual patient profiles, including age, occupation, and smoking status, in managing pneumothorax effectively.

The management and outcomes for SSP highlight the critical role of underlying pulmonary conditions, specifically the significant association between COPD and SSP, as corroborated by studies including Onuki et al. [11]. This relationship underscores the necessity for tailored therapeutic approaches that align with guidelines from authoritative bodies such as the Global Initiative for COPD [26]. These guidelines emphasize individualized management strategies, reflecting the complexity and severity of SSP cases which often necessitate more aggressive interventions like intercostal tube insertion and surgical measures. Despite the higher surgical intervention rates in the PSP group as reported by Onuki et al., the intricate nature of SSP due to co-existing lung pathologies presents a unique challenge, potentially limiting surgical options for some patients [11].

The treatment modalities for SSP vary based on the pneumothorax size and patient's condition, with initial management ranging from observation for small, asymptomatic cases to more invasive interventions like chest tube insertion and surgery for larger or symptomatic pneumothoraces. The BTS and ACCP offer divergent recommendations on the use of NA and ICD insertion, reflecting ongoing debate and evolving evidence on the most effective approaches [20,21]. Surgical intervention remains the cornerstone for reducing recurrence risk and managing PALs, with techniques such as bullectomy, pleurectomy, and chemical pleurodesis being pivotal. Despite advancements in minimally invasive surgeries like VATS, considerations around recurrence rates, patient comorbidities, and procedural tolerability necessitate a balanced decision-making process. Recent literature, including Menassa et al. and a retrospective cohort study by Noda et al., continues to refine our understanding of SSP management, highlighting the importance of integrating surgical advancements with conventional treatment strategies to optimize patient outcomes [27,28].

Our research offers valuable insights into the characteristics and outcomes of patients with PSP and SSP, emphasizing the importance of individualized patient care. The distribution of pneumothorax occurrence (left, right, and bilateral) in both PSP and SSP groups aligns closely with previous studies by Riveiro-Blanco et al. and Onuki et al., suggesting a higher prevalence of right-sided pneumothoraces in SSP patients [4,11]. This pattern may reflect underlying anatomical or physiological differences between PSP and SSP conditions, underscoring the need for tailored diagnostic and treatment approaches.

Furthermore, the recurrence rates observed in our study highlight the significant risk of pneumothorax recurrence, particularly in SSP patients, which is consistent with findings from Riveiro-Blanco et al. and Menassa et al. [4,27]. The association of recurrence with factors such as the presence of blebs/bullae, pleural thickening, absence of active intervention, and prolonged ICD duration underscores the complexity of managing pneumothorax and the potential benefits of surgical intervention over conservative management. Additionally, the LOS and in-hospital mortality rates further differentiate PSP from SSP, with SSP patients experiencing longer hospitalizations and a higher mortality rate, likely due to more severe underlying conditions and the necessity for more invasive treatments.

These findings resonate with the current emphasis on precision medicine, as advocated by the European Respiratory Society [9], highlighting the necessity for clinicians to adopt customized management strategies that consider the unique characteristics of each patient. Our study contributes to the growing body of evidence supporting the need for specialized care pathways for PSP and SSP patients, aiming to improve outcomes, reduce recurrence rates, and minimize postoperative complications. This approach not only enhances patient care but also aligns with global efforts towards more personalized and effective healthcare delivery.

It's essential to acknowledge that our study has limitations, including its retrospective nature, single-center setting, and potential biases inherent in retrospective data collection. Future research could employ prospective multicenter studies to validate these findings across diverse patient populations. Additionally, investigating long-term outcomes and quality-of-life measures in PSP and SSP patients could provide a more comprehensive understanding of their experiences. Future research could explore potential age-specific risk factors contributing to the observed age distribution patterns. Additionally, investigating the role of gender-related factors in pneumothorax etiology and outcomes could provide a more comprehensive understanding of the demographic landscape of these conditions.

## Conclusions

This study highlights the critical importance of distinguishing between primary spontaneous pneumothorax (PSP) and secondary spontaneous pneumothorax, enabling clinicians to deliver more effective, patient-centered care by tailoring interventions to mitigate risks and improve outcomes. It reaffirms that tobacco smoking is a primary risk factor for PSP, with marijuana and cannabis use also contributing to an increased risk of spontaneous pneumothorax (SP). Addressing prolonged air leaks and minimizing recurrence risk stand out as central objectives in SP treatment, with surgical intervention identified as the most effective strategy to prevent recurrence.

## References

[1] Huang Y, Huang H, Li Q (2014). Approach of the treatment for pneumothorax. J Thorac Dis.

[2] Chen N, Zhou M, Dong X (2020). Epidemiological and clinical characteristics of 99 cases of 2019 novel coronavirus pneumonia in Wuhan, China: a descriptive study. Lancet.

[3] Massongo M, Leroy S, Scherpereel A (2014). Outpatient management of primary spontaneous pneumothorax: a prospective study. Eur Respir J.

[4] Riveiro-Blanco V, Pou-Álvarez C, Ferreiro L (2022). Recurrence of primary spontaneous pneumothorax: associated factors. Pulmonology.

[5] Cardillo G, Ricciardi S, Rahman N, Walker S, Maskell NA (2019). Primary spontaneous pneumothorax: time for surgery at first episode?. J Thorac Dis.

[6] Chen JS, Chan WK, Tsai KT (2013). Simple aspiration and drainage and intrapleural minocycline pleurodesis versus simple aspiration and drainage for the initial treatment of primary spontaneous pneumothorax: an open-label, parallel-group, prospective, randomised, controlled trial. Lancet.

[7] Ichinose J, Nagayama K, Hino H, Nitadori J, Anraku M, Murakawa T, Nakajima J (2016). Results of surgical treatment for secondary spontaneous pneumothorax according to underlying diseases. Eur J Cardiothorac Surg.

[8] Baumann MH (2006). Management of spontaneous pneumothorax. Clin Chest Med.

[9] Tschopp JM, Bintcliffe O, Astoul P (2015). ERS task force statement: diagnosis and treatment of primary spontaneous pneumothorax. Eur Respir J.

[10] Hobbs BD, Foreman MG, Bowler R (2014). Pneumothorax risk factors in smokers with and without chronic obstructive pulmonary disease. Ann Am Thorac Soc.

[11] Onuki T, Ueda S, Yamaoka M (2017). Primary and secondary spontaneous pneumothorax: prevalence, clinical features, and in-hospital mortality. Can Respir J.

[12] Bense L, Eklund G, Wiman LG (1987). Smoking and the increased risk of contracting spontaneous pneumothorax. Chest.

[13] Hedevang Olesen W, Katballe N, Sindby JE (2017). Cannabis increased the risk of primary spontaneous pneumothorax in tobacco smokers: a case-control study. Eur J Cardiothorac Surg.

[14] Chen CH, Liao WC, Liu YH (2012). Secondary spontaneous pneumothorax: which associated conditions benefit from pigtail catheter treatment?. Am J Emerg Med.

[15] Baumann MH, Strange C, Heffner JE (2001). Management of spontaneous pneumothorax: an American College of Chest Physicians Delphi consensus statement. Chest.

[16] Asano H, Ohtsuka T, Noda Y, Kato D, Mori S, Nakada T, Matsudaira H (2019). Risk factors for recurrence of primary spontaneous pneumothorax after thoracoscopic surgery. J Thorac Dis.

[17] Walker SP, Bibby AC, Halford P, Stadon L, White P, Maskell NA (2018). Recurrence rates in primary spontaneous pneumothorax: a systematic review and meta-analysis. Eur Respir J.

[18] Noppen M, De Keukeleire T (2008). Pneumothorax. Respiration.

[19] Primavesi F, Jäger T, Meissnitzer T (2016). First episode of spontaneous pneumothorax: CT-based scoring to select patients for early surgery. World J Surg.

[20] MacDuff A, Arnold A, Harvey J (2010). Management of spontaneous pneumothorax: British Thoracic Society Pleural Disease Guideline 2010. Thorax.

[21] Nava GW, Walker SP (2022). Management of the secondary spontaneous pneumothorax: current guidance, controversies, and recent advances. J Clin Med.

[22] Vuong NL, Elshafay A, Thao LP (2018). Efficacy of treatments in primary spontaneous pneumothorax: A systematic review and network meta-analysis of randomized clinical trials. Respir Med.

[23] Erez D, Israeli-Shani L, Epstein Shochet G, King DA, Abu-Akel M, Dovrish Z, Shitrit D (2020). Clinical and radiological characteristics of patients diagnosed with spontaneous pneumothorax: treatment options and clinical outcomes. A retrospective analysis 2004 to 2017. Isr Med Assoc J.

[24] Sadikot RT, Greene T, Meadows K, Arnold AG (1997). Recurrence of primary spontaneous pneumothorax. Thorax.

[25] Brown SG, Ball EL, Macdonald SP, Wright C, McD Taylor D (2014). Spontaneous pneumothorax; a multicentre retrospective analysis of emergency treatment, complications and outcomes. Intern Med J.

[26] (2024). Global Initiative for Chronic Obstructive Lung Disease strategy for the diagnosis, management and prevention of chronic obstructive pulmonary disease: an Asia-Pacific perspective. Respirology.

[27] Menassa M, Malthaner RA, Nayak R (2023). Contemporary interventions for secondary spontaneous pneumothoraces. Shanghai Chest.

[28] Noda M, Watanabe T, Matsuda Y, Sakurada A, Hoshikawa Y, Okada Y (2016). Awake thoracic surgery versus chemical pleurodesis for intractable secondary spontaneous pneumothorax. Surg Today.

